# Incapacitated, Incarcerated Patients: Who Should Decide for Them?

**DOI:** 10.1093/geroni/igaf122.200

**Published:** 2025-12-31

**Authors:** Anurima Chattopadhyay, Daniel Karel

**Affiliations:** National Institutes of Health, Bethesda, Maryland, United States; National Institutes of Health, Bethesda, Maryland, United States

## Abstract

The carceral healthcare system is reckoning with a rapidly aging patient population. This increase poses significant challenges because elderly patients are much less likely to be able to make their own medical decisions, requiring a surrogate decision-maker in about 70% of cases. Currently, prison officials–such as wardens or prison guards–are involved in making medical decisions for about 50% of decisionally incapacitated, incarcerated patients. In contrast, twelve states prohibit prison officials from serving as surrogates for incarcerated patients. This discrepancy highlights the need to more thoroughly examine which parties are appropriate surrogates for incarcerated patients. We consider what qualities make an appropriate surrogate decision-maker. They would, ideally: care about the patient, not have conflicts of interest, and know the patient’s interests and wishes or be otherwise trained to make surrogate decisions. We ultimately determine that prison officials do not best fit these criteria. We then explore alternative approaches that better protect both incarcerated patients and prison officials. We advocate for efforts to raise the rate of advance medical planning amongst incarcerated individuals; currently less than 1% of incarcerated individuals have filled out an advance directive. Healthcare teams should also work to involve incarcerated patients’ families (when appropriate) by addressing issues with security and travel that may prevent them from serving as surrogates. Lastly, when incarcerated patients have no available surrogates, we suggest healthcare teams rely on the relevant guidelines for non-incarcerated, ‘unrepresented’ patients, where decision-making often requires consensus amongst multiple physicians and the involvement of an ethics committee.

